# Supplementary material for: Creating an atlas of the bone microenvironment during oral inflammatory-related bone disease using single-cell profiling
Source: eLife. 2023 Feb 1;12:e82537. doi: 10.7554/eLife.82537 (PMC9925051; doi:10.7554/eLife.82537)
Supplement: Supplementary file 1. [file elife-82537-supp1.docx]

**Supplementary file 1. qRT-PCR primer sequences**

| **Primer name** | **Forward primer** | **Reverse primer** |
| --- | --- | --- |
| Human  *SPARC* | TGAGGTATCTGTGGGAGCTAATC | CCTTGCCGTGTTTGCAGTG |
| Human  *COL3A1* | GGAGCTGGCTACTTCTCGC | GGGAACATCCTCCTTCAACAG |
| Human  *SPP1* | CTCCATTGACTCGAACGACTC | CAGGTCTGCGAAACTTCTTAGAT |
| Human  *OCN* | CACTCCTCGCCCTATTGGC | CCCTCCTGCTTGGACACAAAG |
| Human  *RUNX2* | GGAGTGGACGAGGCAAGAGTTT | AGCTTCTGTCTGTGCCTTCTGG |
| Mouse  *Sparc* | ACCCCCGGCAATTTCATGG | TGTCTTCCCAGCTCTTGATGTAA |
| Mouse  *Runx2* | TCCACAAGGACAGAGTCAGATTACAG | CAGAAGTCAGAGGTGGCAGTGTCATC |
| Mouse  *Col1a1* | GCGCTAAAGGTGCCAATG | AGCACCAGGTTCACCACTG |
| Mouse  *Col1a2* | TCGTGCCTAGCAACATGCC | TTTGTCAGAATACTGAGCAGCAA |
| Mouse  *Bglap* | CTGACCTCACAGATCCCAAGC | TGGTCTGATAGCTCGTCACAAG |
